# Short-Term Sensorimotor Deprivation Impacts Feedforward and Feedback Processes of Motor Control

**DOI:** 10.3389/fnins.2020.00696

**Published:** 2020-07-01

**Authors:** Cécile R. Scotto, Aurore Meugnot, Géry Casiez, Lucette Toussaint

**Affiliations:** ^1^Université de Poitiers, Université de Tours, Centre National de la Recherche Scientifique, Centre de Recherches sur la Cognition et l’Apprentissage, Poitiers, France; ^2^Université Paris-Saclay CIAMS, Orsay, France; ^3^CIAMS, Université d’Orléans, Orléans, France; ^4^Univ. Lille, CNRS, Centrale Lille, UMR 9189 – CRIStAL – Centre de Recherche en Informatique, Signal et Automatique de Lille, Lille, France; ^5^Institut Universitaire de France (IUF), Paris, France

**Keywords:** immobilization, sensorimotor deprivation, Fitts’ task, speed-accuracy trade-off, motor control

## Abstract

Sensory loss involves irreversible behavioral and neural changes. Paradigms of short-term limb immobilization mimic deprivation of proprioceptive inputs and motor commands, which occur after the loss of limb use. While several studies have shown that short-term immobilization induced motor control impairments, the origin of such modifications is an open question. A Fitts’ pointing task was conducted, and kinematic analyses were performed to assess whether the feedforward and/or feedback processes of motor control were impacted. The Fitts’ pointing task specifically required dealing with spatial and temporal aspects (speed-accuracy trade-off) to be as fast and as accurate as possible. Forty trials were performed on two consecutive days by Control and Immobilized participants who wore a splint on the right arm during this 24 h period. The immobilization modified the motor control in a way that the full spatiotemporal structure of the pointing movements differed: A global slowdown appeared. The acceleration and deceleration phases were both longer, suggesting that immobilization impacted both the early impulse phase based on sensorimotor expectations and the later online correction phase based on feedback use. First, the feedforward control may have been less efficient, probably because the internal model of the immobilized limb would have been incorrectly updated relative to internal and environmental constraints. Second, immobilized participants may have taken more time to correct their movements and precisely reach the target, as the processing of proprioceptive feedback might have been altered.

## Introduction

The impact of sensory deprivation has been largely studied to identify behavioral and neural changes following irreversible sensory loss. Over the past decade, such changes have been studied for sensorimotor loss through paradigms involving short-term limb immobilization. Such paradigms mimic the deprivation of motor inputs and outputs that induce maladaptive neural plasticity without compromising brain function (disease-free model; Furlan et al., 2016). Short-term immobilization consists of preventing a body part (often fingers, hand, and/or arm) from moving by means of a splint or a bandage for a period ranging from a few hours to a few days. While several studies have shown that short-term immobilization induced motor control impairments, the origin of such modifications is an open question. Here, a short-term limb immobilization paradigm was used to specify the impact of this sensorimotor deprivation on performance in a Fitts’ task. This pointing task specifically required dealing with spatial and temporal aspects (speed-accuracy trade-off) to be as fast and as accurate as possible. The associated kinematic analysis allowed us to assess whether the feedforward and/or the feedback processes of motor control were impacted.

Overall, studies investigating the anatomical cerebral changes following limb immobilization agree on reductions in cortical excitability of the sensorimotor representation linked to the decrease in sensory input and motor output (Facchini et al., 2002; Huber et al., 2006; Avanzino et al., 2011; Burianová et al., 2016). In the same vein, behavioral studies have highlighted the negative immobilization-induced effects on the cognitive level of action. Alterations at the sensorimotor representation level evaluated by means of an implicit motor imagery task were reported following a few hours of arm non-use (Toussaint and Meugnot, 2013; Debarnot et al., 2018). The authors showed that motor imagery processes used to identify the laterality of hand images were slowed down for stimuli corresponding to the immobilized hand. Other studies reported changes in the peripersonal space representation (Bassolino et al., 2012; Toussaint et al., 2018). Using a reachability judgment task, Toussaint et al. reported that the maximum distance at which objects are perceived as reachable was reduced in subjects forced into arm and hand non-use for 24 h. Overall, these studies have shown that representations in the brain are modified with immobilization.

Although functional consequences of immobilization have been demonstrated, these studies did not identify which mechanisms of action were altered. The majority of studies have tested how short-term immobilization impacted out-and-back uncorrected movements toward visual targets (Huber et al., 2006; Moisello et al., 2008; Debarnot et al., 2018). The hand path trajectory of such movements deviated after immobilization, showing that spatial parameters were impacted (Huber et al., 2006; Moisello et al., 2008). Debarnot et al. (2018) added that temporal parameters were also modified during this out-and-back movement following immobilization. They showed that movement time and reaction time were longer than those of Control participants. While these studies demonstrated the impact of immobilization on spatial parameters on the one hand and temporal parameters on the other hand, the associated kinematic analysis was not provided. This analysis was provided in Bassolino et al. (2012) with a reach-to-grasp objects task. The authors showed that the transport phase was impaired following 10 h of immobilization but not the grasping component (Bassolino et al., 2012). To suppress the possible interaction of the grasping component on the transportation phase, we investigated how short-term immobilization impacts the kinematics of a pointing movement (i.e., without a grasping component). A Fitts’ task was used to specifically assess how immobilization may modify spatiotemporal aspects of motor control. Therefore, contrary to previously used paradigms with immobilization, the Fitts’ task necessitates dealing with speed as well as accuracy (i.e., speed-accuracy trade-off) to reach the target. A kinematic analysis was provided to determine whether the feedforward and/or feedback processes of motor control of the pointing movement were affected. The feedforward model refers to the initiation of early adjustments based on the comparison between the motor commands and the expected outputs (efference copy; Miall and Wolpert, 1996). This feedforward process would be associated with early kinematic parameters (i.e., before peak velocity; Meyer et al., 1982; Elliott et al., 2010). The feedback process corresponds to the correction phase, with an online sensory processing comparing the intended to the current state, and would be associated with later kinematic parameters (i.e., after peak velocity; Meyer et al., 1982; Elliott et al., 2010).

## Materials and Methods

### Participants

Forty-nine right-handed participants (29 men and 20 women; mean age ± SE: 20.0 ± 0.28 years) gave written informed consent prior to the study, in accordance with the 1964 Declaration of Helsinki. The experimental protocol was approved by the ethics committee for research in sciences of physical and sports activities (n°2017250114). All participants reported having normal or corrected-to-normal vision and no neurological or sensorimotor disorders. As we expected, the immobilization effects would disappear within a few trials (Bassolino et al., 2012), and we used a between-subject design. The participants were assigned into one of the two groups (e.g., *Control* or *Immobilized*) and performed either the task with an Index of Difficulty (ID) of *3* or *7* (see section “Procedure”). Fifteen participants constituted the *Control-ID3* group (seven men and eight women; 20.4 ± 0.49 years), twelve were in the *Control-ID7* group (seven men and five women; 20.6 ± 0.49 years), ten were in the *Immobilized-ID3* group (six men and four women; 19.4 ± 0.33 years), and twelve were in the *Immobilized-ID7* group (nine men and three women; 19.6 ± 0.41 years).

### Apparatus

The pointing task was performed on a MacBook Pro Retina (OS X 10.11.6 El Capitan 2.5 GH Core i5) with a screen of 13.3 inches (900 × 1440 pixels) refreshed at 60 Hz. This laptop included an 8.6 cm × 10.5 cm trackpad with a resolution of 400 CPI sampling at 125 Hz. Instructions, stimuli and data from the pointing device were handled using a custom-built application written in C++ using Qt and Libpointing (Casiez et al., 2011). The gain between the trackpad and the visual cursor was set to 1: what was seen on the screen corresponded to what was done on the trackpad.

### Procedure

**FIGURE 1:**
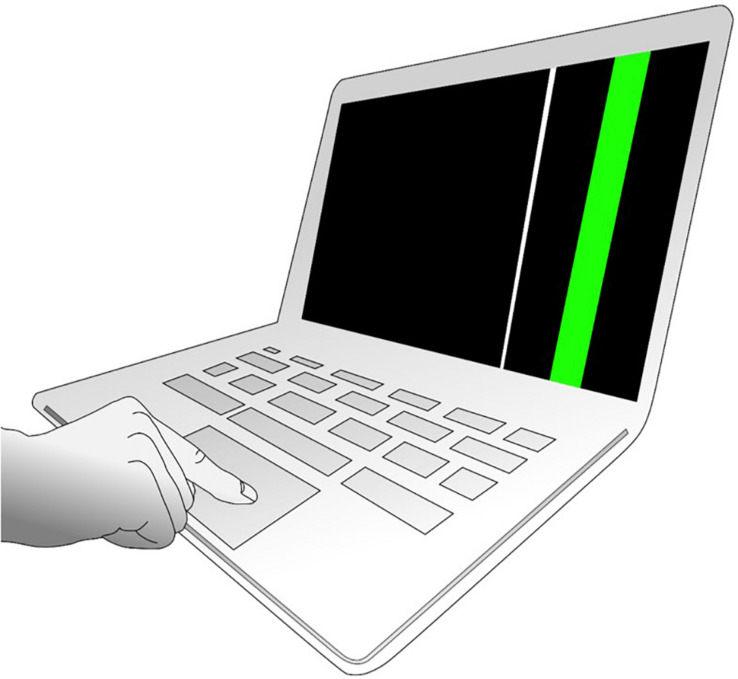
Experimental setup. Subjects were asked to perform a pointing task: They slided their right index on the laptop trackpad to move the visual cursor (white line) into the target (green rectangle).

The task consisted of horizontal 2D pointing (either left to right or right to left; Figure 1) using Fitts’ paradigm (Fitts, 1954). The cursor corresponded to a vertical line of 1 pixel width (0.2 mm), and the target was a rectangle of a length corresponding to the screen height and a width (W), which was manipulated with the task’s ID. The ID integrates both the W and the Distance (D) from the starting point to the target’s center as follows: *ID* = Log2(2D/W). Here, D was set to 8 cm, and W was either 2 or 0.125 cm, defining an ID of 3 or 7, respectively. The participants in the two groups (*Control* and *Immobilized*) were assigned to either *ID3* or *ID7* conditions (between-subject design; see section “Participants”).

In an illuminated room, the participants sat in a chair adjustable in elevation. They were approximately at 50 cm faced to a laptop. The experimenter placed their forearm on the table perpendicular to the laptop, in a comfortable position. The joints were not restrained, and the pointing mainly consisted of a wrist movement (i.e., abduction when pointing from the left and adduction when pointing from the right). Talcum was applied on the participant’s finger before the experiment to reduce dampness and allowed an easy finger slide on the trackpad. This talcum was reapplied whenever the participants needed it. For each trial, the participants were instructed to explore the trackpad with the right finger to find the starting position on the trackpad (left or right border). When the position was reached, the word “calibration” was displayed on the screen. The trial was launched if the finger was static at this specific location for 0.5 s. The trial started with the simultaneous appearance of the cursor and the visual target: The participants could then start the pointing whenever they were ready. The required movement was to point the visual target as precisely and as accurately as possible with a smooth and continuous movement on the trackpad. The participants had to avoid stopping before or after the target. After a period of 0.5 s static in the target, the visual stimuli disappeared, and the trial stopped. The trial direction alternated between rightward and leftward. The vision of the arm was not restrained.

The experiment was composed of two sessions of 40 trials on 2 consecutive days (*Pre* and *Post* tests). The first session (*Pre*) also included a training of 20 trials to familiarize the participants with the task prior to data recording. The *Pre* session lasted 40 min. Immediately after this first session, the participants in the *Immobilized* group had their right arm immobilized with a rigid splint (DONJOY “Comfort Digit”; DJO, Surrey, United Kingdom) that firmly maintained the wrist and three fingers (index, middle, and ring). An immobilization vest (model DONJOY “Immo Axmed”) restraining right shoulder, arm and forearm movements was also used to ensure that the participants kept their arm at rest as much as possible during the 24 h of immobilization. The *Immobilized* group also wore actimeters (ActiGraph wGT3X-BT) on the wrist of both hands to verify if they had complied with these instructions. The actimeters recorded the activity level (in counts/min) with ActiLife software (ActiLife v6.11.8, Pensacola, FL, United States).

Twenty-four hours after the first session, both groups returned and performed the second session of 40 trials (*Post*). For the *Immobilized* group, the *Post* test was performed immediately after splint removal by the experimenter. The *Post* session lasted 15 min.

### Data Processing

For the Immobilized group, a quantitative check of the activity amount was performed through the recording of both arms with actimeters. During the 24 h immobilization period, 638 ± 59 counts/min were recorded for the right immobilized hand and 2795 ± 115 counts/min were recorded for the left non-immobilized hand (see Toussaint and Meugnot, 2013 for a similar procedure). ANOVA performed on the actimeter values confirmed that the level of manual activity was higher for the left hand than for the right hand [*F*_(1,21)_ = 525.5; *p* < 0.001].

Position data from the trackpad were low-pass filtered with a dual-pass, no-lag Butterworth filter (cutoff frequency: 10 Hz; order: 2). The data were then derivated to compute the finger velocity used to determine the Movement Time (MT) of the pointing. The MT corresponds to the period between the movement onset and offset, which were defined when the velocity reached above and below 5% of Peak Velocity (PV), respectively. We further assessed the impact of immobilization with the analysis of pointing corrections. Although the participants were instructed to point the target with a “*smooth and continuous movement*,” some movements were stopped (velocity below 5% of PV) before or after the target. We computed the percentage of trials where corrections appeared (i.e., the correction rate). Movement kinematics were also analyzed to further determine the impact of immobilization on the motor *impulse phase* and online *correction phase*, associated to feedforward and feedback processes, respectively. Modifications in the *impulse phase* were assessed through the analysis of the time of acceleration (AT; time from movement onset to PV). In addition, the time of deceleration (DT; time between PV and movement offset) was associated with the *correction phase* with online corrections (Meyer et al., 1982; Elliott et al., 2010). DT includes the deceleration period of the first submovement (from PV) as well as the period lasting for all potential additional submovements.

Bassolino et al. (2012) found that the influence of immobilization in a reach-to-grasp task did not last more than a few trials. Therefore, we first assessed whether differences appeared over trials by comparing the means of each eight successive blocks of five trials. Repeated-measures ANOVAs were then conducted using a mixed design with two between-subjects factors: *Group* (*Control* vs. *Immobilized*) and *ID* (3 vs. 7) and two within-subjects factors: *Session* (*Pre* vs. *Post*) and *Block* (1–8). A simple effect of Block appeared but no significant interactions were revealed between the *Block* and the *Group* or the *Session* on all the dependent variables. The analyses were then conducted on the mean of the 40 trials. *Post-hoc* tests (Newman-Keuls) were performed when necessary, and the level of significance was set at 0.05 for all statistical analyses.

## Results

### Kinematic Profiles

**FIGURE 2:**
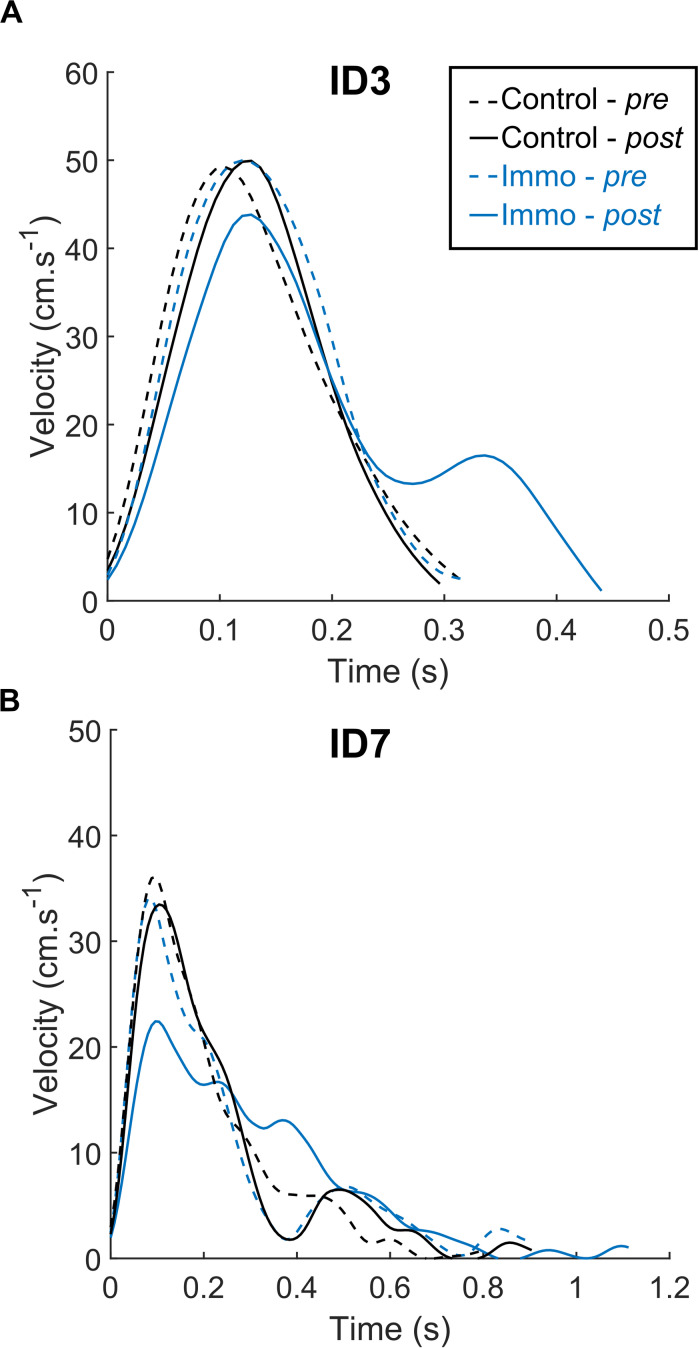
Velocity profiles from representative trials for the *Control* and *Immobilized* (*Immo*) participants who performed in either the *ID3* **(A)** or *ID7* **(B)** condition. Dashed lines represent session *Pre*, and full lines represent session *Post*. Regardless of the *ID*, the *Immobilized* participants exhibited longer MT and lower PV in the *Post* session than in the *Pre* session.

Figure 2 depicts velocity profiles for representative trials in the Control and Immobilized groups. As classically shown, the *ID* seemed to modify pointing kinematics: *ID7* was associated with a lower PV, longer MT and more corrections than *ID3*. In addition, Figure 1 suggests that the *Immobilized* participants exhibited a longer MT and a lower PV in the *Post* session than in the *Pre* session, regardless of the ID. These observations were statistically tested with mean comparisons of selected kinematic parameters.

### Movement Time and Corrections

**FIGURE 3:**
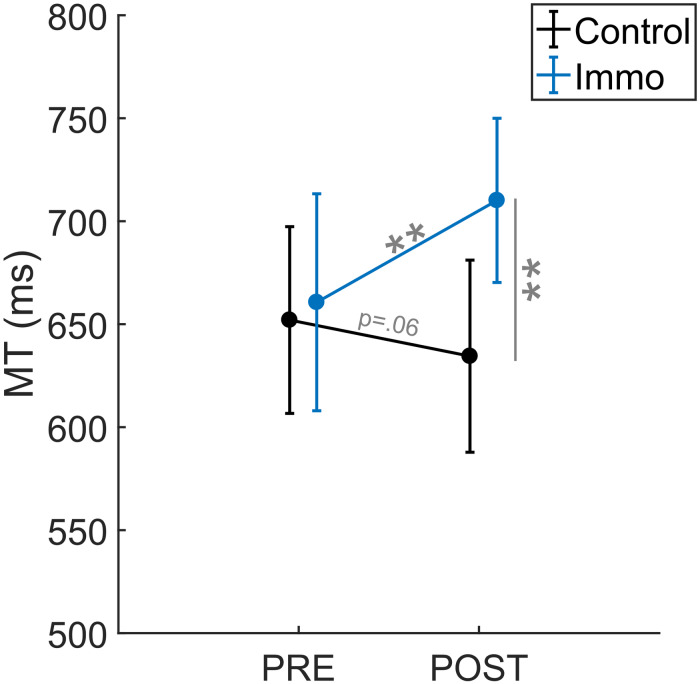
Movement Time (MT) relative to the Session (*Pre* vs. *Post*) for the *Control* and *Immobilized* (*Immo*) groups. **p < 0.01. Error bars denote standard error.

The repeated-measures ANOVA *Group* × *ID* × *Session* for MT revealed an effect of *ID* [*F*_(1,45)_ = 349.0; *p* < 0.001] as well as an interaction *Group* × *Session* [*F*_(1,45)_ = 10.4; *p* < 0.001]. Overall, the MT was shorter in the *ID3* than in the *ID7* condition (367 ± 12 ms vs. 970 ± 32 ms, respectively). No significant effect of Group [*F*_(1,45)_ = 1.6; *p* = 0.21], Session [*F*_(1,45)_ = 2.4; *p* = 0.13], Group × ID [*F*_(1,45)_ = 0.0; *p* = 1.00], Session × ID [*F*_(1,45)_ = 0.2; *p* = 0.62] nor Group × ID × Session [*F*_(1,45)_ = 2.0; *p* = 0.17] appeared. Figure 3 depicts the *Group* × *Session* interaction. The *Control* and *Immobilized* groups differed between the *Pre* and *Post* sessions. For the *Immobilized* group, the MT increased between the *Pre* and *Post* sessions (661 ± 70 ms vs. 710 ± 73 ms, respectively; *p* < 0.01). In contrast, for the *Control* group, the MT did not increase between the *Pre* and *Post* sessions and either exhibited a trend toward a decrease (666 ± 64 ms vs. 639 ± 62 ms; *p* = 0.06).

The analysis of corrections (under- and overshoots) revealed an effect of ID [*F*_(1,45)_ = 26.9; *p* < 0.001] with a higher correction rate for ID7 than ID3 (2.0 ± 0.6% vs. 14.1 ± 2.5%). No significant effect of Group [*F*_(1,45)_ = 0.9; *p* = 0.35], Session [*F*_(1,45)_ = 0.1; *p* = 0.70], Group × ID [*F*_(1,45)_ = 0.5; *p* = 0.50], Session × ID [*F*_(1,45)_ = 0.0; *p* = 0.95], Session × Group [*F*_(1,45)_ = 0.0; *p* = 0.87] nor Group × ID × Session [*F*_(1,45)_ = 0.7; *p* = 0.42] appeared. Therefore, the analysis failed to show an effect of immobilization on corrections.

### Acceleration and Deceleration Time

**FIGURE 4:**
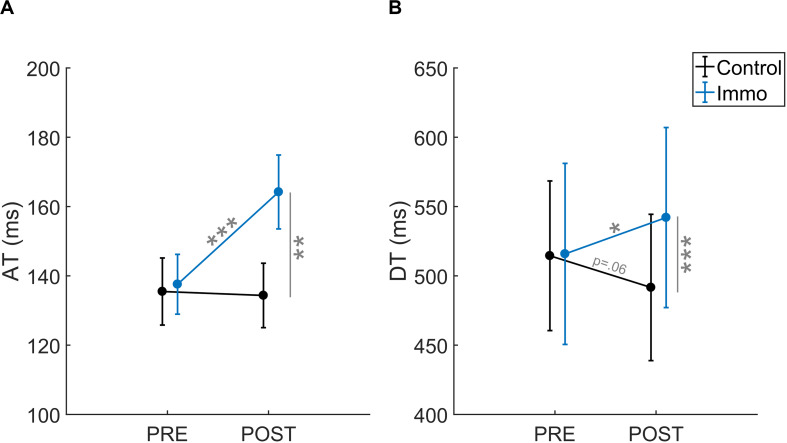
**(A)** Acceleration Time (AT) and **(B)** Deceleration Time (DT) relative to the Session (*Pre* vs. *Post*) for the *Control* and *Immobilized* (*Immo*) groups. *p < 0.05; **p < 0.01; ***p < 0.001. Error bars denote standard error.

The Acceleration Time (AT) corresponds to the absolute period between the movement onset and the PV. This parameter is associated with the impulse phase of motor control reflecting the planning process of the movement. The analysis showed an effect of *ID* [*F*_(1,45)_ = 34.6; *p* < 0.001], *Session* [*F*_(1,45)_ = 9.7; *p* < 0.01] and a *Group* × *Session* interaction [*F*_(1,45)_ = 11.5; *p* < 0.01]. No significant effect of Group [*F*_(1,45)_ = 2.5; *p* = 0.19], Group × ID [*F*_(1,45)_ = 0.1; *p* = 0.73], Session × ID [*F*_(1,45)_ = 0.9; *p* = 0.35] nor Group × ID × Session [*F*_(1,45)_ = 1.4; *p* = 0.25] appeared. Therefore, the AT was shorter at *ID3* (113 ± 4 ms) than at *ID7* (172 ± 11 ms). Moreover, the *post-hoc* analysis of the interaction showed that the *Control* and *Immobilized* groups differed between the *Pre* and *Post* sessions (Figure 4A). For the *Immobilized* group, the AT increased between the *Pre* and *Post* sessions (138 ± 9 ms vs. 164 ± 11 ms, respectively; *p* < 0.001). For the *Control* group, the AT did not increase between the *Pre* and *Post* sessions (136 ± 10 ms vs. 134 ± 9 ms; *p* = 0.86).

The Deceleration Time (DT) corresponds to the absolute period between the PV and the end of the movement. This parameter is associated with the homing phase of motor control reflecting online movement corrections (e.g., Woodworth, 1899; Meyer et al., 1982). The analysis showed an effect of ID [*F*_(1,45)_ = 553.1; *p* < 0.001] and a *Group* × *Session* interaction [*F*_(1,45)_ = 9.3; *p* < 0.01]. No significant effect of Group [*F*_(1,45)_ = 1.2; *p* = 0.27], Session [*F*_(1,45)_ = 0.0; *p* = 0.83], Group × ID [*F*_(1,45)_ = 0.6; *p* = 0.43], Session × ID [*F*_(1,45)_ = 0.0; *p* = 0.88] nor Group × ID × Session [*F*_(1, 45)_ = 0.7; *p* = 0.42] appeared. Again, the DT was shorter at ID3 (242 ± 9 ms) than at ID7 (790 ± 23 ms), and *post-hoc* analysis of the interaction showed that the *Control* and *Immobilized* groups differed between the *Pre* and *Post* sessions (Figure 4B). For the *Immobilized* group, the DT increased between the *Pre* and *Post* sessions (516 ± 65 ms vs. 542 ± 65 ms, respectively; *p* < 0.05). For the *Control* group, the DT did not increase between the *Pre* and *Post* sessions and exhibited a trend toward a reduction (514 ± 54 ms vs. 492 ± 53 ms; *p* = 0.06).

Further analyses were conducted to determine what caused the modifications in the temporal parameters (i.e., MT, AT, DT). More precisely, we computed the peak acceleration, velocity and deceleration to determine whether those modifications occurred at an early or late stage.

### Peak Velocity, Peak Acceleration, and Peak Deceleration

**FIGURE 5:**
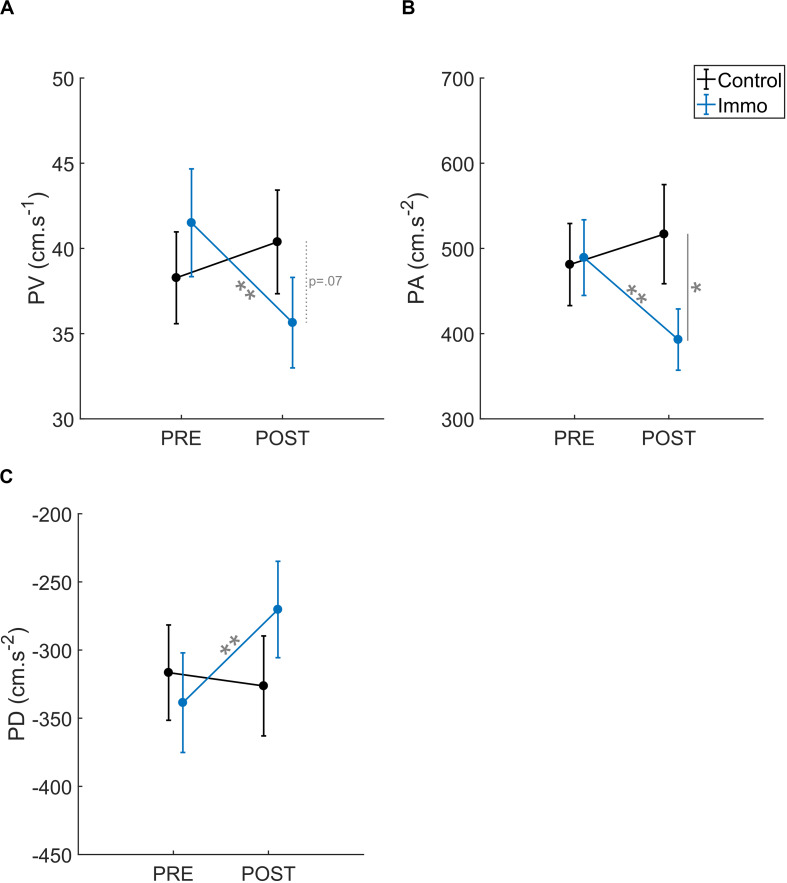
**(A)** Peak Velocity (PV), **(B)** Peak Acceleration (PA), and **(C)** Peak Deceleration (PD) relative to the Session (*Pre* vs. *Post*) for the *Control* and *Immobilized* (*Immo*) groups. *p < 0.05; **p < 0.01. Error bars denote standard error.

We analyzed how fast the pointing movements of the participants were. The analysis of Peak Velocity (PV) revealed an effect of *ID* [*F*_(1,45)_ = 66.3; *p* < 0.001] and a *Group* × *Session* interaction [*F*_(1,45)_ = 13.3; *p* < 0.01; Figure 5A]. No significant effect of Group [*F*_(1,45)_ = 0.1; *p* = 0.77], Session [*F*_(1,45)_ = 3.0; *p* = 0.09], Group × ID [*F*_(1,45)_ = 0.4; *p* = 0.53], Session × ID [*F*_(1,45)_ = 0.5; *p* = 0.50] nor Group × ID × Session [*F*_(1,45)_ = 0.3; *p* = 0.57] appeared. First, the PV was higher at ID3 (49.5 ± 2.0 cm.s^–1^) than at ID7 (28.4 ± 1.9 cm.s^–1^). In addition, the *post-hoc* analysis of the interaction showed that the PV in the *Immobilized* group decreased between the *Pre* and *Post* sessions (41.5 ± 3.2 cm.s^–1^ vs. 35.6 ± 2.7 cm.s^–1^, respectively; *p* < 0.01). No difference was found for the *Control* group between the *Pre* and *Post* sessions (514 ± 2.7 cm.s^–1^ vs. 492 ± 3.0 cm.s^–1^; *p* = 0.06). A trend appeared in the *Post* session between the two groups (*p* = 0.07).

The analysis of Peak Acceleration (PA) revealed an effect of ID [*F*_(1,45)_ = 52.6; *p* < 0.001] and a *Group* × *Session* interaction [*F*_(1,45)_ = 10.3; *p* < 0.01; Figure 5B]. No significant effect of Group [*F*_(1,45)_ = 1.6; *p* = 0.21], Session [*F*_(1,45)_ = 2.2; *p* = 0.15], Group × ID [*F*_(1,45)_ = 2.0; *p* = 0.16], Session × ID [*F*_(1,45)_ = 1.6; *p* = 0.21] nor Group × ID × Session [*F*_(1,45)_ = 0.0; *p* = 0.90] appeared. As with the PV, the PA was higher at ID3 (634 ± 40 cm.s^–2^) than at ID7 (306 ± 31 cm.s^–2^). In addition, the *post-hoc* analysis of the interaction showed that the PA in the *Immobilized* group decreased between the *Pre* and *Post* sessions (489 ± 44 cm.s^–2^ vs. 393 ± 36 cm.s^–2^, respectively; *p* < 0.01). No difference was found for the *Control* group between the *Pre* and *Post* sessions (481 ± 48 cm.s^–2^ vs. 517 ± 58 cm.s^–2^; *p* = 0.19). Finally, the *Pre* and *Post* sessions differed between the two groups (*p* < 0.01).

The analysis of Peak Deceleration (PD) revealed an effect of ID [*F*_(1,45)_ = 57.6; *p* < 0.001] and a *Group* × *Session* interaction [*F*_(1,45)_ = 6.4; *p* < 0.01; Figure 5C]. No significant effect of Group [*F*_(1,45)_ = 0.3; *p* = 0.61], Session [*F*_(1,45)_ = 3.6; *p* = 0.06], Group × ID [*F*_(1,45)_ = 0.8; *p* = 0.37], Session × ID [*F*_(1,45)_ = 1.0; *p* = 0.31] nor Group × ID × Session [*F*_(1,45)_ = 0.1; *p* = 0.72] appeared. As PA, the PD was also higher at ID3 (−438 ± 29 cm.s^–2^) than at ID7 (−188 ± 21 cm.s^–2^). In addition, the *post-hoc* analysis of the interaction showed that the PD in the *Immobilized* group decreased between the *Pre* and *Post* sessions (−339 ± 37 cm.s^–2^ vs. −270 ± 35 cm.s^–2^, respectively; *p* < 0.05). No difference was found for the *Control* group between the *Pre* and *Post* sessions (−317 ± 35 cm.s^–2^ vs. −326 ± 37 cm.s^–2^; *p* = 0.60) or between the *Pre* and *Post* sessions between the two groups (*p* = 0.14).

## Discussion

Here, we tested how short-term immobilization modified behavioral responses. More precisely, we tested whether the feedforward and/or feedback processes of pointing movements were affected by 24 h of arm non-use. We first showed that immobilization had an impact on sensorimotor control with lengthened movement time without damaging accuracy. This decrease in movement time seemed to result from a global slowdown: The acceleration and deceleration phases were both longer and were associated with lower peak acceleration, velocity, and deceleration. Therefore, immobilization appeared to modify sensorimotor control in such a way that the full spatiotemporal structure of the pointing movements differed.

First, our data confirmed that immobilization leads to a decrease in motor performance (Huber et al., 2006; Moisello et al., 2008; Bassolino et al., 2012; Bolzoni et al., 2012). Such declines in pointing performance have been shown to arise from changes in joint coordination around the deprived segment (Moisello et al., 2008; Bassolino et al., 2012). When the immobilized participants were instructed to make out-and-back straight movements without correction, an increase in the hand-path area amplitude and variability appeared (Huber et al., 2006; Moisello et al., 2008; Bassolino et al., 2012). When the task integrated spatial constraints for trial validation, immobilization rather induced temporal impairments, such as an increase in movement time. Therefore, Bassolino et al. (2012) showed an increase in movement time for a reach-to-grasp task where spatial constraints were defined (i.e., the object reaching movement to perform the grasping) during the five first trials. Here we found that this increase in movement duration could last longer for a pointing task as we did not find an interaction between the immobilization and the trial repetitions. Therefore, no reactivation of the process of proprioceptive inputs would appear contrary to the results of Bassolino et al. (2012). Albeit, here the movement amplitude was reduced and required a less complex motor control than a reach-to-grasp task involving multiple joints from the arm, the hand and the digits. In addition, spatial corrections (stops before or after the target) were not amplified with immobilization, as Fitts’ paradigm requires finishing the movement in the target position, and we instructed the participants to perform a “*smooth movement*.” Therefore, the participants would lengthen their movement rather than doing several sub-movements to reach the target. In addition, we hypothesized that the behavioral consequences of immobilization would be modulated by task difficulty. Therefore, we expected a higher impact with ID7 than ID3 because motor planning and control is more complex. While we found the classic effect of ID on the kinematic parameters, no statistical interaction appeared with the immobilization factor. The lack of proprioceptive cues would be sufficiently strong to affect any movement, as also suggested by the work of Medina et al. (2009) on a deafferented patient (JDY). In this study, the difference in movement time between controls and JDY would not appear to be modulated by the tested ID (i.e., 4, 4.5, and 5.5) of the pointing task.

Before debating what immobilization changed for feedforward and/or feedback processing, we have to discard the possibility that changes arising from peripheral structures declined. Indeed, immobilization leads to modifications in muscle contractile properties (from slow to fast fiber type) and motor units. However, such transformations appeared after several weeks of immobilization (Desaphy, 2001; Seki et al., 2001a, b; Zanette et al., 2004). In contrast, short-term immobilization (less than 3–4 days) do not impact muscle and nerve properties (Facchini et al., 2002; Huber et al., 2006; Moisello et al., 2008). Therefore, modifications of motor behavior in the present study cannot be attributed to changes in muscle structure following short-term immobilization.

Here, we showed that immobilization impacted both early and late movement kinematics. Since Woodworth’s two-component model, kinematic parameters before the peak velocity are associated with *feedforward control* and those after are associated with the *feedback control* (Meyer et al., 1982; Elliott et al., 2010). On the one hand, we showed that immobilization lengthened acceleration duration as well as decreased peak acceleration. Early kinematics modifications have been shown to reflect the use of internal models, i.e., a representation of the action and its sensory consequences (e.g., future states of the arm at the end of pointing; Wolpert et al., 1995). Based on these feedforward inputs of the limb, predictions of the future states are compared to the current state which allow for early corrections (Wolpert et al., 1995; Desmurget and Grafton, 2000; Wolpert and Ghahramani, 2000). Our results suggested that the feedforward control was impacted, probably because the internal model of the immobilized limb would be incorrectly updated. In daily life, the internal model of the limb is continuously updated through motion (see Wolpert and Ghahramani, 2000). During the 24 h of immobilization, motor commands of the limb were largely reduced. Consequently, efference copy as well as dynamical proprioceptive cues could not have been used to maintain or calibrate the internal model with the limb dynamics relative to the environment. Such a decrease in feedback would lead to an altered prediction of the sensory consequences of the action before its execution. Studies with deafferented patients have previously shown that proprioception was critical to update internal models of limb dynamics (Sainburg et al., 1995; Sarlegna et al., 2006; Medina et al., 2009).

On the other hand, our results suggested that immobilization also modifies the feedback control of the pointing: A lengthened deceleration duration as well as decreased peak deceleration were observed. These results suggested disruptions in the process of proprioceptive cues correcting the movement online. This is in line with recent studies (Huber et al., 2006; Weibull et al., 2011; Ngomo et al., 2012; Rosenkranz et al., 2014; Opie et al., 2016), which found a decrease in excitability in the somatosensory areas representing the previously immobilized arm: The proprioceptive cues were less processed, as well as the tactile cues (i.e., decrease of tactile discrimination; Weibull et al., 2011). Therefore, immobilized participants would take more time to correct their movement to precisely reach the target, as the processing of proprioceptive cues might be altered. Visual cues would be particularly used to compensate for this deficit, notably with the online visual comparison between the cursor and the target position. This feedback control of the pointing movement throughout vision was shown to start later than the proprioceptive one (Sarlegna et al., 2004; Saunders and Knill, 2004), which could explain the increase of the correction phase duration. This hypothesis is supported by neurophysiological data which showed that the decrease in cortical excitability of the somatosensory areas of the immobilized limb is associated with a sensitivity increase of the other sensory inputs (Rosenkranz et al., 2014). Further experiments would be necessary to specifically isolate how visual cues impact sensorimotor control after immobilization.

Although functional consequences of immobilization have been demonstrated in the past, the impact of immobilization on the motor control processes has not been fully elucidated. Contrary to previously used paradigms with immobilization, we used a Fitts’ task which necessitates dealing with spatiotemporal constraints (i.e., speed-accuracy trade-off). Thanks to a spatiotemporal kinematic analysis, we specifically assessed the impact of sensorimotor deprivation on the motor control processes. For the first time, we showed early and late kinematic changes following a short period of limb non-use, which may be caused by the modification of feedforward as well as feedback processes. Even if these results would have to be extended to a broader population, such as the elderly people, they may have implications in rehabilitation and health care. Everybody has been or will be immobilized during his/her own lifetime due to an accident (e.g., broken limb) or for external reasons (e.g., long travel, prolonged bed rest). The understanding of the sensorimotor consequences of such short-term immobilization thus appeared of particular interest.

## Data Availability Statement

The datasets generated for this study are available on request to the corresponding author.

## Ethics Statement

The studies involving human participants were reviewed and approved by the ethics committee for research in sciences of physical and sports activities (n°2017250114). The patients/participants provided their written informed consent to participate in this study.

## Author Contributions

CS, AM, and LT conceived and designed the research. CS and GC set up the experiments. LT performed the experiments. CS analyzed the data and prepared the figures. CS and LT interpreted the results of the experiments and drafted the manuscript. CS, AM, LT, and GC edited and revised the manuscript. All authors contributed to the article and approved the submitted version.

## Conflict of Interest

The authors declare that the research was conducted in the absence of any commercial or financial relationships that could be construed as a potential conflict of interest.
